# Pregnancy-related acute myocardial infarction after treatment with ritodrine hydrochloride: A case report

**DOI:** 10.1016/j.crwh.2026.e00804

**Published:** 2026-03-22

**Authors:** Naofumi Yamane, Maiko Sagawa, Sayaka Yamada, Hikari Sanada, Shina Harimoto, Yumiko Kan, Megumi Tsunakake, Hiroko Nakamura, Masatoshi Kumagai

**Affiliations:** a Department of Obstetrics and Gynecology, Hiroshima University Hospital, Hiroshima, Japan; b Department of Obstetrics and Gynecology, National Hospital Organization Kure Medical Center, Hiroshima, Japan

**Keywords:** Pregnancy-associated myocardial infarction, Pregnancy complications, Ritodrine hydrochloride, Vasospastic angina, Case report

## Abstract

Pregnancy-associated myocardial infarction (PAMI) is a rare but potentially life-threatening condition for the mother and fetus. Pregnancy also confers a three- to fourfold increased risk of MI compared with the non-pregnant state. However, recognizing PAMI promptly in obstetric settings remains challenging.

A 34-year-old Japanese primigravida at 30 weeks of gestation had been receiving ritodrine for preterm labor. On hospital day 2, approximately 4 h after discontinuation of tocolytics, repeat electrocardiography (ECG) indicated new ST-segment elevation in leads I, aVL, and V3-V6 without chest pain. High-sensitivity troponin I was 5392 pg/mL, CK-MB was 21 IU/L, and transthoracic ECG revealed mild apical hypokinesis. That night, prolonged fetal bradycardia prompted emergency Cesarean delivery. Maternal cardiac biomarkers then declined rapidly, and the ECG and wall-motion abnormalities resolved. Myocardial perfusion scintigraphy (day 5) and coronary computed tomography angiography (day 6) showed no perfusion defect or obstructive coronary disease. The infant, weighing 1546 g, was admitted to the neonatal intensive care unit. No adverse effects attributable to ritodrine were observed, and the infant was discharged on postnatal day 58.

The report describes probable drug-induced PAMI temporally associated with β₂-agonist tocolysis, with plausible mechanisms including transient coronary vasospasm and supply-demand mismatch in the hemodynamic milieu of pregnancy. This case suggests that women receiving β₂-agonist tocolysis should be monitored for vital signs, electrolytes, and ECG changes. Even without chest pain, new ECG abnormalities or palpitations warrant immediate cardiac evaluation.

## Highlights

• In the reported case silent myocardial infarction occurred during ritodrine tocolysis for threatened preterm labor.
• Electrocardiography, echocardiography, and high-sensitivity troponin identified myocardial injury.
• Careful cardiac monitoring should be considered when using ritodrine in pregnancy.

## 1 Introduction

Acute myocardial infarction (AMI) in pregnancy and the puerperium is uncommon, but carries a substantial maternal and perinatal risk. Pregnancy induces profound hemodynamic, prothrombotic, and hormonal changes that cause a three- to fourfold increase in the risk of MI compared with the non-pregnant state [1]. The incidence is in the order of a few to 10 cases per 100,000 deliveries [2]. Population-based data and contemporary series have clarified the epidemiology and etiologies, including atherosclerotic plaque rupture, spontaneous coronary artery dissection (SCAD), and coronary vasospasm, and emphasize the need for rapid diagnosis and multidisciplinary care [2], [3]. This report describes a case of pregnancy-associated myocardial infarction (PAMI) following exposure to ritodrine hydrochloride, discusses plausible mechanisms, including vasospasm, and reviews diagnostic and management considerations relevant to obstetric practice.

## 2 Case Presentation

A 34-year-old Japanese primigravida (G1P0) with a history of incomplete right bundle branch block (IRBBB) conceived with timed intercourse. She took no regular medications, had no known allergies, and denied a family history of cardiovascular disease and smoking. At 29 + 2 weeks of gestation, irregular uterine contractions with cervical shortening were noted at a referring hospital, and oral ritodrine hydrochloride (2 tablets/day) was initiated. At 30 + 1 weeks, she was admitted for worsening contractions and started continuous intravenous ritodrine. Intramuscular betamethasone sodium phosphate was also administered to a cumulative dose of 24 mg. At 30 + 3 weeks, despite up-titration of ritodrine to 166 μg/min, contractions remained difficult to suppress. Intravenous magnesium sulfate (1 g/h) was added and the patient was moved to a tertiary perinatal center.

**Fig. 1:**
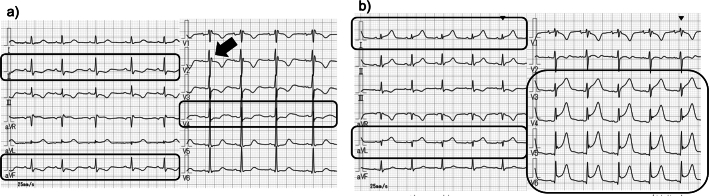
Electrocardiograms on arrival (a) and hospital day 2 (b). (a) Incomplete right bundle branch block (arrow) with ST-T depression in leads II, aVF, and V4 (boxed). (b) New ST-segment elevation in leads I, aVL, and V3-V6 (boxed), consistent with acute myocardial ischemia. Boxes indicate regions of interest. Abbreviations: ECG, electrocardiogram; IRBBB, incomplete right bundle branch block.

On arrival, the cervix was 1 cm dilated with painless contractions every 6–8 min. The patient was hemodynamically stable (heart rate 89 bpm; blood pressure 125/59 mmHg), although she reported palpitations. Laboratory tests indicated leukocytosis (white blood cell count 20,200/μL) and elevated C-reactive protein (10.0 mg/dL) with hypokalemia (K 3.2 mEq/L). Electrocardiography (ECG) showed baseline IRBBB, a prolonged QT interval of 497 ms, and ST-T depression in leads II, aVF, and V4 (Fig. 1a). In view of the inflammatory markers, chorioamnionitis was suspected.

On hospital day 2, all tocolytics were discontinued given concern for chorioamnionitis. During the following 4 h, occasional tachycardia in the 90–120 bpm range was observed, although the patient remained hemodynamically stable. Approximately 4 h after discontinuation of tocolytics, repeat ECG revealed new ST-segment elevation in leads I, aVL, and V3-V6 (Fig. 1b). At that time, her vital signs were stable (heart rate 90 bpm; blood pressure 98/66 mmHg), and she reported palpitations without chest pain. Serum studies revealed mild transaminitis (AST 46 IU/L, ALT 25 IU/L), myocardial injury (CK-MB 21 IU/L [reference ≤12 IU/L], and high-sensitivity troponin I 5392 pg/mL [reference ≤26 pg/mL]). Transthoracic echocardiography (TTE) showed mild hypokinesis of the apical segments, raising a strong suspicion for AMI (Table 1). Ritodrine-associated, drug-induced PAMI was included in the differential diagnosis. Because tocolytics had already been discontinued, maternal vital signs were stable, and the wall-motion abnormality was mild, a conservative strategy was chosen, with close cardiology collaboration and serial TTE.

**Table 1:** Laboratory findings on hospital day 2.

[Complete Blood Count]			[Biochemistry & Cardiac biomarkers]		[Coagulation]		
WBC	20200 /μL		AST	46 IU/L	PT-INR	1.01	
Neut	92.2 %		ALT	25 IU/L	APTT	25.5 sec	
Lymph	3.4 %		LDH	180 IU/L	D-dimer	2.2 mg/dL	
Mono	4.3 %		BUN	35.2 mg/dL			
Hb	13.7 g/dL		Cr	0.38 mg/dL			
Hct	27.3 %		Na	136 mEq/L			
Plt	22.1 ×10^4^/μL		Cl	104 mEq/L			
			K	3.6 mEq/L			
			Mg	3.2 Mg/dL			
			CK-MB	321 IU/L			
			Troponin I	5392 pg/mL			
			NT-proBNP	25 pg/mL			

That evening, spontaneous labor ensued. During ultrasound monitoring, fetal bradycardia lasting >2 min occurred, prompting emergency Cesarean section. A male neonate (weight 1546 g) was delivered with Apgar scores of 7 and 8 at 1 and 5 min, respectively. The umbilical arterial pH was 7.300. The infant was admitted to the neonatal intensive care unit (NICU) due to the low birth weight. No neonatal adverse effects attributable to ritodrine, such as tachycardia or hypokalemia, were observed. The infant was discharged from the NICU on day 58 of life.

**Fig. 2:**
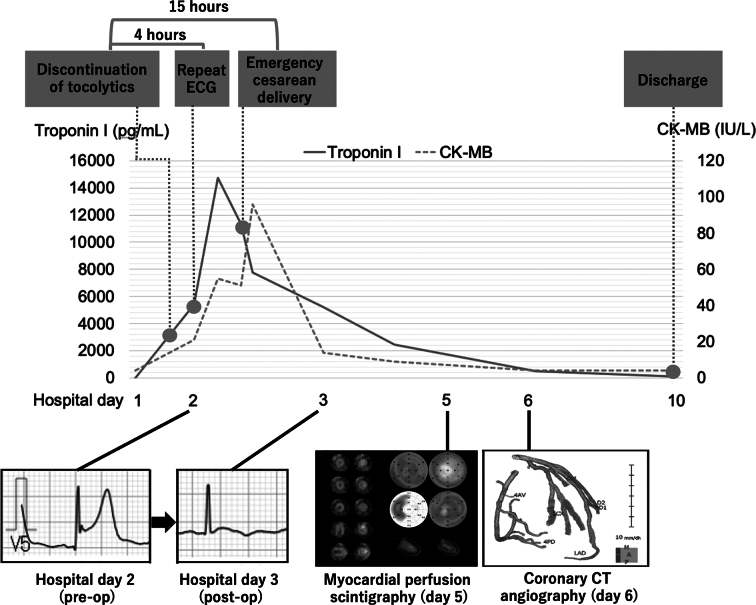
In-hospital clinical course. ECG abnormalities and a rise in cardiac biomarkers were detected on hospital day 2. Later that night, prolonged fetal bradycardia necessitated emergency Cesarean *sec*tion. Postoperatively, maternal hemodynamics stabilized and ventricular function improved, permitting discharge on hospital day 10.

Postoperatively, maternal cardiac biomarkers declined rapidly, and the ECG abnormalities and apical hypokinesis resolved. Myocardial scintigraphy and cis obtained preoperatively was negative. Placental histopathology showed no chorioamnionitis, funisitis, or placental abruption. Given the temporal association with β₂-agonist exposure, the ischemic ECG/TTE findings with biomarker elevation, and the absence of obstructive coronary disease or myocarditis, ritodrine-related drug-induced PAMI was considered highly likely. The postoperative course was uneventful and the patient was discharged on postoperative day 7 (hospital day 10) with a plan for periodic cardiology follow-up (Fig. 2).

## 3 Discussion

Cardiovascular conditions in pregnancy, including peripartum cardiomyopathy, PAMI, pulmonary embolism, and myocarditis, often present with chest pain or dyspnea and can be challenging to diagnose [1]. PAMI is defined as MI occurring in pregnancy or puerperium. Although uncommon (2–10 cases per 100,000 deliveries), the maternal and fetal mortality rates in PAMI are about 7% and 5%, respectively, and multidisciplinary management is frequently required [2], [4]. Pregnancy itself increases the risk of AMI by three to four times, and onset is most common from late pregnancy to the postpartum period [2], [5]. Sudden hemodynamic and hormonal shifts may provoke endothelial dysfunction and thrombosis, contributing to the pathogenesis [1], [3]. As in non-pregnant patients, most cases present with chest pain, ECG changes, biomarker elevation, and regional wall-motion abnormalities in the corresponding coronary territory [1], [2].

Atherosclerosis predominates as the usual etiology of MI, whereas there is a higher frequency of SCAD in PAMI. Therefore, coronary angiography and CCTA are important for defining the mechanism and guiding management in pregnant and postpartum patients [1], [3]. With appropriate precautions, including short fluoroscopy time, a restricted field, filtration, and abdominal shielding, fetal radiation in these diagnostic procedures can be kept well below thresholds associated with harm, and urgent evaluation should not be deferred when clinically indicated [4], [5].

Drug exposure has also been implicated in PAMI. Ergot derivatives used postpartum and the β₂-agonist ritodrine hydrochloride, as in the present case, have both been suggested as potential precipitants [2], [6], [7], [8]. Beyond case-based observations, pharmacoepidemiologic data indicate a short-term increase in major adverse cardiovascular events after initiation of inhaled β₂-agonists [9]. Physiological changes in pregnancy, such as the increase in plasma volume and elevation of cardiac output, compounded by β₂-agonist-induced tachycardia, may raise myocardial oxygen demand and shorten diastole, thereby reducing coronary perfusion and precipitating supply-demand mismatch [1].

In the present case, ischemia persisted after discontinuation of ritodrine. This drug has an elimination half-life of 2–3 h, and thus its pharmacologic effects can continue for several hours after cessation [10]. β₂-agonists dose-dependently increase heart rate and lower serum potassium, changes that can reduce the ischemic threshold; therefore, with higher doses, resolution of ischemia may be delayed even after the drug is withdrawn [11]. Coronary vasospasm can also persist or recur despite removal of a provoking factor (e.g., a drug trigger), a pattern consistent with drug-induced PAMI; and vasospastic events during β₂-agonist therapy have been described in respiratory medicine [6], [8], [12]. β₂-agonists may not directly induce spasm, but tachycardia and heightened endogenous noradrenaline can prolong or exacerbate vasospasm [6]. A similar mechanism may have contributed to the course in the present case.

The adverse reactions of ritodrine include cardiovascular complications and electrocardiographic abnormalities such as ST-segment depression and QT-interval prolongation [11], [13]. Accordingly, patients receiving ritodrine should undergo regular ECG surveillance. When abnormalities are detected, prompt assessment with cardiac biomarkers (e.g., CK-MB and troponin) and transthoracic ECG is warranted to evaluate for myocardial injury. If cardiac dysfunction or ongoing ischemia is suspected, early collaboration with cardiologists is needed to determine additional testing and management. Because non-specific ECG changes are common in pregnancy, findings should be interpreted in conjunction with symptoms such as palpitations, chest pain, and fatigue.

## 4 Conclusion

PAMI associated with β₂-agonist tocolysis may be clinically silent. Therefore, obstetric teams should maintain a high index of suspicion not only when peripartum chest symptoms occur, but also when new maternal tachycardia, palpitations, electrolyte abnormalities, or ECG changes are detected. Such findings should prompt early multidisciplinary evaluation with ECG, cardiac biomarkers, and echocardiography, with coronary angiography considered when clinically indicated.

## Contributors

Naofumi Yamane contributed to patient care, conception of the case report, acquiring and interpreting the data, drafting the manuscript, undertaking the literature review and revising the article critically for important intellectual content.

Maiko Sagawa contributed to patient care, conception of the case report, acquiring and interpreting the data and revising the article critically for important intellectual content.

Sayaka Yamada contributed to revising the article critically for important intellectual content.

Hikari Sanada contributed to revising the article critically for important intellectual content.

Shina Harimoto contributed to revising the article critically for important intellectual content.

Yumiko Kan contributed to patient care, acquiring the data and revising the article critically for important intellectual content.

Megumi Tsunakake contributed to revising the article critically for important intellectual content.

Hiroko Nakamura contributed to patient care, acquiring the data and revising the article critically for important intellectual content.

Masatoshi Kumagai contributed to revising the article critically for important intellectual content.

All authors approved the final submitted manuscript.

## Patient consent

Written informed consent was obtained from the patient for publication of the case report and accompanying images.

## Provenance and peer review

This article was not commissioned and was peer reviewed.

## Funding

No funding from an external source supported the publication of this case report.

## Declaration of competing interest

The authors declare that they have no competing interest regarding the publication of this case report.
